# Registered dietitians’ beliefs and behaviours related to counselling patients on physical activity and sedentary behaviour from a theory of planned behaviour perspective

**DOI:** 10.1186/s40795-020-00392-1

**Published:** 2020-11-30

**Authors:** Jessica Huntington, John J. M. Dwyer, Sara Shama, Paula Brauer

**Affiliations:** Department of Family Relations and Applied Nutrition, University of Guelph, Macdonald Institute Building, 50 Stone Road East, Guelph, Ontario N1G 2W1 Canada

**Keywords:** Qualitative, Physical activity, Sedentary, Dietitian, Counsel, Theory of planned behaviour

## Abstract

**Background:**

Registered dietitians working in team-based primary care settings (e.g., family health teams [FHTs]) are positioned to counsel on physical activity and sedentary behaviour when providing nutrition-related services to promote health and prevent disease. This qualitative study explored FHT registered dietitians’ beliefs and behaviours related to counselling patients on physical activity and sedentary behaviour.

**Methods:**

Twenty registered dietitians in FHTs in Ontario, Canada were interviewed in person. Theory of planned behaviour guided the development of this cross-sectional, descriptive study. Thematic analysis was used to identify themes within each of the following theoretical constructs (topics): registered dietitians’ behaviour (practice), behavioural intention, attitude, subjective norm, and perceived behavioural control related to physical activity and sedentary behaviour counselling.

**Results:**

All participants counselled patients on physical activity, using some motivational interviewing strategies, and most counselled on sedentary behaviour. Many participants intended to continue their current physical activity counselling practices and increase sedentary behaviour counselling. Some participants had a positive attitude about the effectiveness of counselling on physical activity and sedentary behaviour, but their belief about effectiveness was dependent on factors such as time frame for behaviour change. Many participants felt that other health care professionals expected them to counsel on physical activity and they believed that other registered dietitians counsel on physical activity and sedentary behaviour. Facilitators to counselling included FHT dynamics and time with patients. In terms of barriers, almost all participants were confident in basic PA counselling only and contended that only this is within their scope of practice. Many participants posited that exercise prescription is outside their scope of practice. Other barriers included registered dietitians’ lack of knowledge and not having a physical activity expert on the team.

**Conclusions:**

The results suggest that strategies are warranted to improve FHT registered dietitians’ knowledge, attitude, and counselling skills related to physical activity and sedentary behaviour. This study provides a strong foundation to develop a theory-based, quantitative measure to assess physical activity and sedentary behaviour counselling practices and determinants among registered dietitians.

**Supplementary Information:**

The online version contains supplementary material available at 10.1186/s40795-020-00392-1.

## Background

The World Health Organization [1] defines physical activity (PA) as any movement of the body that expends energy and is produced by skeletal muscles. The current Canadian guidelines state that adults should participate in at least 150 min of moderate-to-vigorous intensity PA (MVPA) per week in at least 10-min bouts [2]. The term “physically inactive” is typically used to describe individuals who fail to meet the minimum recommendations for PA. An evaluation of previous systematic reviews found that regular PA reduces the risk of at least 25 chronic health conditions (e.g., hypertension, cardiovascular disease, type 2 diabetes, and some types of cancers) and it can be beneficial for both primary and secondary prevention of these conditions [3]. Also, PA can be used as a strategy to promote mental health [4]. A Canadian national study of adults during 2016–2017 indicated that, based on accelerometer data, adults accumulated an average of 26 min of MVPA daily (total of 10-min bouts and sporadic PA) and only 16% of adults met the Canadian MVPA guidelines that include 10-min bouts [5].

Conversely, sedentary behaviour (SB) is any waking activity in which energy expenditure is low (≤ 1.5 metabolic equivalents, a standardized unit for measuring the intensity of PA in research) while sitting or lying down [6]. There is insufficient research to provide a recommended limit for adults’ total SB time during the day and a guideline for how often SB should be interrupted with PA [7]. High levels of SB have been shown to increase the risk of cardiovascular disease-related mortality and all-cause mortality [8, 9]. An evaluation of past systematic reviews has shown an association between high levels of SB among adults and increased risk of cardiovascular disease, metabolic syndrome, hypertension, depression, type 2 diabetes, and some cancers (ovarian, colon and endometrial) [8]. Increased levels of SB can have negative health outcomes even in the presence of adequate levels of PA [9]. Statistics Canada [10] (a national statistical office) reported that, based on accelerometer data collected during 2016–2017, Canadian adults accumulated an average of 567 min of SB per day.

Promotion of PA continues to be needed. Less than 15% of Canadians are aware of the PA guidelines [11]. A positive relationship has been observed between awareness of the PA guidelines and meeting the guidelines [11], suggesting a need for increased awareness. Physicians and other health care professionals (HCPs) are in an ideal position to increase PA and SB awareness and improve lifestyle behaviours by integrating PA and SB counselling into their daily practice [12, 13]. Previous literature indicates that HCPs are the dominant source of health information for many adults [14], particularly for those who do not meet the PA guidelines [15]. PA counselling by HCPs has also been found to be effective in creating behaviour change [12]. Despite this, PA counselling remains underutilized by many HCPs [12, 14, 16].

Although all HCPs could promote PA in their daily practice, registered dietitians (RDs) are in a particularly ideal position to counsel patients on both PA and SB because of their main focus on lifestyle change [17–19]. As previously mentioned, physical inactivity is associated with increased risk of several chronic conditions, most of which are associated with nutrition-related factors as well [20]. While RDs do use a lifestyle approach when counselling patients [21], their focus has traditionally been nutrition, but could include sleep hygiene, PA, and SB.

To date, few studies have examined HCPs’ PA attitude and/or counselling behaviours and most have focused on physicians, nurses, medical students or a mix of HCPs [12, 22–24]. To our knowledge, there have been few studies that have examined PA counselling among RDs [17, 20, 25], two of which were completed more than 10 years ago [17, 25]. Furthermore, there have been no studies that have examined SB counselling among RDs, and only one study has examined SB counselling among HCPs [26]. Also, most studies related to HCPs’ PA counselling practices have used a quantitative approach [17, 20, 23, 25, 27].

In Ontario, a new collaborative team-based model of primary care, family health teams (FHTs), was introduced in the mid-2000s. Various HCPs were included (e.g., family physicians, nurses, nurse practitioners, dietitians, social workers, and pharmacists) but few PA specialists were employed. There are currently 184 FHTs in Ontario [28]. The vision was that FHTs would provide both clinical and preventive care services [29, 30]. Each team decides which services they offer, including PA services. In this study, we qualitatively explore FHT RDs’ beliefs and behaviours related to counselling patients on PA and SB. The study results can inform future training needs of primary care RDs and other HCPs, where PA specialist services are not available.

## Methods

Ethics clearance was obtained from the researchers’ university and written consent was obtained from participants.

### Study design and sampling

This qualitative, cross-sectional, descriptive study was part of a larger research project [31]. RDs employed at least 3 months in a FHT lacking PA specialist services were eligible. A sampling frame of FHTs within a 2-h driving radius of Guelph, Ontario was created. Approximately one third of FHTs with both an RD and PA specialist (whom RDs could refer patients to) were excluded. Study recruitment information was emailed to FHT executive directors to forward to RDs, and the first 20 eligible RDs were selected as participants.

### Theoretical framework

Theory of planned behaviour (TPB) [32] guided the development of this study as well as the subsequent thematic analysis, consistent with the approach used in some previous qualitative health research that was based on TPB [33–35]. Thematic analysis was used to identify themes within each of the TPB constructs. According to TPB [32], behaviour is determined by intention, which is a person’s readiness to perform a specific behaviour. Three main determinants of behavioural intention are attitude, subjective norm, and perceived behavioural control. Attitude is a person’s positive or negative feelings about performing a specific behaviour. Subjective norm is a person’s perception of social pressure to perform a specific behaviour [32], which is based on both injunctive normative beliefs (perception of what significant others expect you to do regarding a specific behaviour) and descriptive normative beliefs (perception of what significant others are doing regarding a specific behaviour) [32, 36]. Perceived behavioural control is a person’s perceived ability to perform a specific behaviour, which is based on beliefs about factors that may facilitate or obstruct performing a specific behaviour [32]. Perceived behavioural control is often viewed as being the same as self-efficacy, which is a person’s belief about having the ability to perform a specific behaviour, especially when encountering various obstacles [36].

### Semi-structured interview guide

Before asking the interview questions, PA and SB were defined as per World Health Organization [1] and Tremblay et al. [6]. The interview guide was comprised of questions developed to examine the TPB constructs (see Supplementary file: Interview Guide). Two questions focused on each of the following constructs: PA and SB counselling practices (behaviour); intention to counsel patients on PA and SB in the future; attitude toward PA and SB counselling; subjective norm regarding PA and SB counselling practices (injunctive and descriptive normative beliefs); and perceived behavioural control to counsel patients on PA and SB (factors that make it easy or difficult to counsel patients on PA and SB). PA and SB counselling was defined as actively discussing PA and SB with patients, including providing education regarding the benefits of increasing PA or reducing SB, discussing PA recommendations, assessing patients’ current PA and SB status, and setting goals with patients regarding their PA and SB.

### Self-administered background questionnaire

The questionnaire to assess participants’ background characteristics included questions about participants’ education, duration of employment in the FHT, age, and self-identified gender.

### Procedure

The first author conducted in-person audio-recorded interviews with the 20 participants at their workplaces to examine behaviour, behavioural intention, attitude, subjective norm, and perceived behavioural control related to PA and SB counselling (which subsequently yielded theoretical saturation). The first author was a graduate student who was trained in qualitative interviewing (e.g., she completed a graduate course in qualitative research methods that included interview training). Each interview was approximately 60 min in duration. Participants also completed the background questionnaire. This study procedure was previously pilot tested with two RDs who were not in the study sample of 20 RDs. Descriptive statistics were conducted on the quantitative, background questionnaire data (using Statistical Package for the Social Sciences [version 24]). Braun and Clarke’s [37] thematic analysis approach was used to analyze qualitative data (using NVivo for Mac [Qualitative Solutions and Research International, 2015]):

1. The first author transcribed the interviews and imported them into NVivo for Mac.
2. The first and third authors familiarized themselves with the data by reading the transcripts and taking notes on preliminary ideas.
3. They identified themes relevant to the research purpose.
4. They identified broader patterns/themes by combining themes.
5. They reviewed these themes to explore relationships between themes.
6. They defined and named themes and identified illustrative quotes.
7. The first author wrote a report of the thematic analysis results.

This comprehensive, systematic approach ensured trustworthiness of the results through credibility, transferability, dependability, and confirmability [38]. Comparing the audio-recorded interviews and the transcripts yielded accurate or credible data [37]. Triangulation (e.g., two authors constantly compared identified themes) and thick description of data (e.g., via an in-depth account of participants’ experiences and perspectives as FHT RDs counselling on PA and SB) established credibility and transferability, respectively [37, 38]. However, in terms of transferability, sampling RDs in FHTs in Ontario and not collecting more detail about RDs’ background characteristics (e.g., their specialty area in nutrition/dietetics) limit the extent to which the results can be generalized to other RDs working in team-based primary care elsewhere. Dependability and confirmability were established through an audit trail [38]. For example, detailed records related to the audio-recorded interviews, transcripts, preliminary ideas written on transcripts, and tables that showed themes with corresponding descriptions of themes and illustrative quotes were kept.

## Results

All participants (*n* = 20) had an undergraduate degree in nutrition/dietetics and many participants also obtained higher levels of education (e.g., Master’s degree, 40%; post-graduate diploma, 5%), 90% were employed in their FHT for 6 months or longer, most participants were 25–34 years of age (25–34 years, 65%; 35–44 years, 15%; 45–54 years, 5%; 55–64 years, 15%), and 19 identified as women and one identified as a man.

To summarize the results of the thematic analysis, the following themes (in parentheses) related to TPB constructs were identified: (a) PA counselling practices [behaviour] (PA versus SB focus in counselling; motivational interviewing strategies and other general strategies); (b) SB counselling practices [behaviour] (SB counselling prompted by high levels of SB); (c) behavioural intention (maintain PA counselling and increase SB counselling); (d) attitude (mixed views about effectiveness of counselling); (e) subjective norm (comprehensive healthy lifestyle counselling; presumption that other RDs counsel on PA and SB); (f) perceived behavioural control: facilitators for PA and SB counselling (dynamics of the FHT; time with patients); and (g) perceived behavioural control: barriers to PA and SB counselling (RDs’ scope of practice; expertise).

### Behaviour

#### RDs’ PA counselling practices

##### PA versus SB focus in counselling

Twelve participants discussed focusing predominantly on PA counselling instead of SB counselling. Reasons for this included the perceived higher level of patient acceptance of PA counselling versus SB counselling and participants’ desire to focus on adding positive lifestyle behaviour rather than eliminating negative lifestyle behaviour.

*I don’t usually address screen time or reducing [SB] because focusing on adding something is usually a lot easier than focusing on removing something. … It’s the same thing as saying cut your food portions back. Usually, we don’t find that as successful as when we say, ‘Could you add one more vegetable on the plate?’ … The focus is on ways we can get [the patient moving] more, as opposed to reducing [SB]. (Participant 8)*

##### Motivational interviewing strategies and other general strategies

All participants used some strategies in motivational interviewing, which is collaborative, patient-centered counselling designed to intrinsically motivate patients to change behaviour [39–41]. They discussed the importance of and their adherence to patient-centered counselling in their practice, including during PA counselling.

*I have a background in motivational interviewing ... so I use that in the work that I do.... I’m not someone who just says ‘Here, follow this meal plan’. It’s more like, ‘What are you doing and how can we move you along with this?’ So it goes along with that whole idea, ‘How do we get you more active? How do I get you less sedentary?’ That fits with ‘How do I get you eating more fruits and vegetables?’ ... So again, it’s all that patient-centered type of thing where the individuals really takes ownership of it and I just have to help motivate them, help support them, and help them with ideas. (Participant 6)*

Motivational interviewing strategies discussed included assessing readiness to change (nine participants), working with patients to identify and overcome barriers (14 participants), and patient-led goal setting (all participants).

*It’s a lot of exploring with them what they are willing to do and having them think about things that they might like versus getting into very detailed specifics.... I don’t recommend specific PA plans but it’s more exploring with them and maybe moving them more towards the preparation stage if they’re at the contemplation stage. (Participant 5)*

*If they’re not doing any PA at the moment, what are some barriers? So from my perspective, I would like to hear a story from every single client before I make an assumption or before I make any recommendations on increasing their PA level. (Participant 12)*

*I cannot put my goals in somebody else’s head. They have to come up with it themselves. That’s crucial with any kind of counselling including nutrition. So definitely my experience in this whole area [of PA counselling] is motivational counselling and getting people to move forward from wherever they’re at and doing that in a positive manner. (Participant 10)*

In terms of other general strategies, almost all participants discussed establishing a baseline level of PA with each patient before counselling on the topic, which involved participants asking patients about their current PA habits.

*I ask all of my patients what PA they engage in because I like to get a baseline of how active or sedentary they are. … I’ll ask what their PA routine is, what their days look like, if they engage in any regular PA or exercise, if they go to the gym, if they wear pedometers, and things like that. (Participant 3)*

Also, some participants encouraged patients to monitor their PA level independently because they felt this was a helpful tool for patients who are trying to increase their PA levels. This included recording PA minutes per day and using fitness trackers or pedometers.

*I have food journals that patients will complete and … on the side, it will have PA ... so they can write down every day how much activity they did … . That gives you an idea of how much activity they’re doing weekly, … and I have had a few clients use a pedometer … . They found it helpful just seeing which days they have more steps and what they were doing that day. (Participant 9)*

As well, many participants discussed providing general education on PA to their patients. Participants discussed educating patients on the benefits of PA and the PA guidelines. Participants promoted walking as a safe and free activity that most people can do to achieve health benefits. Some participants discussed guiding patients to PA resources such as information about local exercise classes or examples of places to obtain affordable exercise equipment.

#### RDs’ SB counselling practices

##### SB counselling prompted by high levels of SB

Sixteen participants reported counselling patients on SB, however the majority (13/16) only did so if high levels of SB were mentioned by the patient or derived from patient information. An example of this is patients who mentioned they had a “desk job” where they were seated at a desk for most of their day. In these situations, participants discussed increasing patients’ awareness of their SB level.

*I think its self-awareness that we need to get at. I don’t think people realize how much they sit, so a chart where they write every half hour that they’re sitting, whether they’re eating, reading, or watching TV, is actually an eye-opener for people. (Participant 13)*

Participants stated that their counselling focused predominantly on small SB reduction strategies such as decreasing or breaking up periods of screen time and adding PA during times that were normally sedentary (e.g., walking at lunch instead of sitting at the desk).

### Behavioural intention

#### Maintain PA counselling and increase SB counselling

Fourteen participants felt that unless something changes in their scope of practice or available resources, they will likely continue their current practices with PA counselling *(*e.g.*, “My intention is to continue bringing PA up and having people include PA in their lives as much as they can to help manage health conditions or prevent any kind of chronic health concerns.” (Participant 1)).*

Sixteen participants intended to increase their SB counselling in the future.

*I think with that new big study about how harmful SB is, it would be great for me to incorporate a discussion about SB in some shape or form into my assessment and go in a little bit more depth rather than it just being a roundabout kind of conversation. If I had clear guidelines [about SB] or I knew what they were, I could definitely incorporate that in. (Participant 20)*

### Attitude

#### Mixed views about effectiveness of counselling

Nine participants felt that RDs’ counselling on PA and SB was effective *(*e.g.*, “Patients who do the walking that I recommend find they have more energy …*. *They may not incorporate my food changes but they incorporate the walking and they really like how it increases their energy level.” (Participant 7)).* The remaining participants were unsure of the effectiveness of RDs counselling on PA and SB or they felt that the effectiveness varied greatly depending on a variety of factors *(*e.g.*, “I think effectiveness really depends on number one, the patient’s motivation, and number two, the dietitian’s ability to respect that patient and do proper motivational counselling.” (Participant 10)).*

Five participants believed that their counselling would help patients change their PA or SB, but the participants did not think that the change would necessarily be sustained, and therefore counselling may not have long-term health benefits.

*I think it can be effective in getting them to think about PA or to learn things that they didn’t know about PA. In terms of actually creating change, depending on how long we’re able to follow a patient, it might have limited effectiveness because if we only see them twice, we can’t really follow up on if they did their PA for the past three weeks, for example. (Participant 15)*

### Subjective norm

#### Comprehensive healthy lifestyle counselling

This theme was identified within the topic of injunctive normative beliefs. Eighteen participants contended that all HCPs, including RDs, should provide general counselling on PA and SB. Participants described general counselling as explaining the benefits of PA, goal setting for PA or SB, encouraging low-intensity PA (e.g., walking), and sharing the PA guidelines. Twelve participants reported that other HCPs expect them to do general PA or SB counselling, but not go into depth on these topics. Participants felt that it was expected that RDs counsel on healthy lifestyle as a whole.

*I think they know that healthy eating and PA go hand-in-hand, so I think they expect me to at least address [PA] to the best of my ability. Often, if I do get a request to see [a patient], the doctor will say, ‘Please discuss lifestyle strategies,’ so it’s not just diet. (Participant 1)*

Conversely, the remaining participants felt that other HCPs thought of RDs as diet experts only and therefore did not expect them to counsel on PA or SB. They asserted that other HCPs might not fully understand or utilize RDs’ ability to counsel patients on lifestyle factors beyond nutrition.

*A lot of physicians, and sometimes the nurses, just think I talk about food. They wouldn’t necessarily come to me and think that I could do [PA or SB counselling] because they just think my education is in food and nutrition. (Participant 6)*

Eight participants specifically asserted that all RDs should counsel patients on PA and SB because of the synergistic nature of PA, SB, and nutrition.

*I think dietitians are definitely in a good position to do [PA and SB counselling] because we have the time. Usually we have an hour set aside for our appointments, so we have time to go into more detail with people about things, and we’re focusing on lifestyle changes anyway, so it often goes hand-in-hand with making diet changes. (Participant 2)*

*It [counselling on PA] is really beneficial because you’re talking about nutrition and setting goals [and] that often goes along with PA. It’s a really natural part of the discussion. I think clients feel comfortable with that as well. (Participant 4)*

#### Presumption that other RDs counsel on PA and SB

This theme was identified within the topic of descriptive normative beliefs. Seventeen participants thought that other RDs in FHTs are likely counselling patients on PA *(*e.g.*, “I think in terms of discussing [PA], everybody would be doing that. It’s easy to link food with activity …. It’s part of being a dietitian.” (Participant 18)).* The remaining few participants were entirely unsure of other FHT RDs’ PA counselling practices.

Thirteen participants thought that other RDs in FHTs are likely counselling patients on SB. Of these participants, three presumed that RDs are only touching on SB indirectly in their counselling.

*I think [other dietitians are counselling on SB] but I think indirectly. I think counselling on SB is a way of getting to people who don’t want to do PA. So if we talk about 30 minutes of PA five days a week and they say ‘I just can’t do that’, I think that’s when most dietitians shift to, ‘Okay, so where can we add movement? And where can we reduce sitting?’ (Participant 13)*

In contrast, only three participants felt that other RDs are not counselling patients on SB. The remaining four participants were largely unsure of other RDs’ SB counselling practices because it was not a topic of conversation among RDs.

### Perceived behavioural control

#### Facilitators

##### Dynamics of the FHT

Eighteen participants stated that they are confident in providing only basic PA counselling (e.g., educating patients about benefits of and guidelines for PA and helping patients set goals) in their FHT. Eight participants discussed how various types of access that they have as a FHT member facilitates their counselling. For example, participants explained how they have access to patients’ interdisciplinary health care charts that provide a fuller picture of their health, which helps in tailoring counselling to each patient’s individual needs. Three participants discussed how the focus of FHTs to prevent chronic disease facilitates their counselling on PA and SB. They elaborated on the unique and relatively new role of RDs in the community setting, as opposed to hospital or clinical settings.

*There’s a whole switch to where dietitians are in the community now as health promoters keeping people out of the hospital …. Maybe that’s part of the whole dietitian’s role now, to encourage less SB and more PA to help keep people out of the hospital, keep them healthy, and help them live longer and have a better quality of life. (Participant 6)*

##### Time with patients

Fifteen participants discussed having ample time during appointments to counsel patients about PA and SB as a facilitator. Participants commented that RD appointments were typically 30–60 min whereas physician appointments were shorter, such as 10–15 min. For example, a participant said *“It’s important that we’ve got the time to do motivational interviewing and find out what’s important to them and understand the barriers.” (Participant 16)).*

#### Barriers

##### RDs’ scope of practice

Eighteen participants contended that only basic PA counselling is within their scope of practice.

*I feel comfortable explaining the theory to people. I feel comfortable and confident in explaining how it [PA] can help benefit chronic disease management and prevention. In terms of more specific instruction, that’s when I don’t feel as confident. Again, it falls outside the scope of practice or I’m not trained in it. (Participant 14)*

Five participants felt their PA counselling was limited because assessing patients’ PA is outside RDs’ scope of practice. Participants felt that it is also inappropriate to recommend or counsel patients on vigorous PA because they felt as RDs, they do not have the knowledge or ability to assess the fitness level of their patients or to safely make recommendations for high-intensity PA. Seven participants felt that demonstrating exercises is outside their scope of practice.

*For our diabetes prevention programs, … we’ll follow along to an exercise video together. That’s where it becomes more tricky because as a dietitian, in our scope of practice, you can’t show people how to do exercises but we can follow along a video together. (Participant 15)*

Twelve participants said that providing detailed exercise prescription is outside their scope of practice. They noted liability issues and increased risk of patient injury as a result of improperly prescribed exercises as concerns. Conversely, five participants stated that they are confident in SB counselling because SB is a simpler concept than PA and that SB counselling (unlike PA counselling) fits within their scope of practice.

Half of participants discussed the importance of referring to other HCPs when they felt the level of PA counselling necessary for a patient was outside their scope of practice. For example, they mentioned referring patients to a doctor if patients had medical conditions that could affect their safety when participating in PA or referring to a physiotherapist when patients needed or desired more advanced counselling.

##### Expertise

Twelve participants discussed their lack of expertise as a barrier, which included lacking knowledge, skills, and/or training to go into more depth with PA counselling. In terms of formal training to become an RD, participants discussed receiving a limited amount of education in PA and PA counselling. They acquired most of their PA knowledge independently. Seven participants elaborated specifically on their limited knowledge about SB and SB counselling *(*e.g.*, “For adults, I was never aware of any strategies [for reducing SB] …. I don’t have a toolbox of strategies to tell people how to reduce their SB.” (Participant 5)).* Nine participants expressed interest in pursuing further education in PA, SB, and counselling in these areas *(*e.g.*, “I haven’t [pursued further education in PA or SB counselling] so far. But if I see something pop up through the Nutrition Resource Center or Dietitians of Canada, I would definitely do it.” (Participant 18)).*

Fourteen participants identified not having a PA expert on the FHT as a barrier, stating that it was difficult to not have an expert available to refer patients to. Thus, participants felt responsible to do PA counselling themselves.

*The difficulty is not having someone more specialized in exercise. It kind of falls on us [RDs] a lot of the time so that can make it a little more challenging …. [Other HCPs will say], ‘If you have a question about exercise, you can see the dietitian’, even though I don’t have any additional training in [PA counselling]. (Participant 2)*

## Discussion

To our knowledge, this is the first study to assess both PA and SB counselling among RDs. Guided by TPB, this study explored FHT RDs’ behaviour, behavioural intention, attitude, subjective norm, and perceived behavioural control (facilitators and barriers) related to PA and SB counselling. A main finding was that all participants counselled patients on PA, which is similar to results from previous quantitative research [17, 20, 25]. All participants in the current study used some strategies in motivational interviewing. Motivational interviewing is a multi-faceted, patient-centered, and goal-oriented form of counselling focused on increasing intrinsic motivation among patients and creating sustainable behaviour change [39–41]. This approach includes working with patients to assess their readiness to change, resolve their uncertainties, and ultimately allow ideas for change to come from the patient and not the counselor [39–41]. Patient-centered counselling is defined as providing optimal care for patients that is based on patients’ personal values, needs, and preferences and stresses the importance of involving patients in all decision-making processes regarding their care [42]. Regarding counselling strategies, George et al. [20] found that 30% of RDs assessed patient readiness when counselling patients on PA, a theme that was more prevalent in the current study (nearly half of participants addressed readiness). Furthermore, though other studies regarding RDs did not specifically discuss the prevalence of motivational interviewing for PA counselling, researchers found that the majority of RDs had training in motivational interviewing [20, 25]. Previous literature indicates that motivational interviewing can be effective when used in PA counselling or other health behaviour counselling [43–46], including dietary counselling by RDs [47]. Based on the main themes identified in the current study, one could posit that participants utilized a counselling style that is similar to the 5A framework (ask, advise, assess, assist, and arrange) [48] for PA counselling even though they did not use that term. To illustrate, each construct in this framework with possible corresponding themes from the present study is as follows: ask (establish a PA baseline), advise (general PA education), assess (assess readiness to change), assist (goal setting; guide patients to resources), and arrange (refer to other HCPs). This framework is widely used in counselling for other health behaviours, such as smoking cessation [49] and weight-loss counselling [50]. The amount of time that current participants spent counselling individual patients on PA was not discussed, but PA counselling should involve multiple sessions for sustained behaviour change. A systematic review of the effectiveness of PA counselling in primary care, specifically patient counselling by family physicians or teams, indicated that a single session focusing on a discussion of the patient’s motivation may increase PA 1 year later but follow-up counselling may sustain this PA level after 1 year [43].

More studies are needed to further understand SB counselling and prevalence. Most participants reported counselling patients on SB. In contrast, a previous study that assessed SB counselling among physicians found that, based on patients’ reports, 10% of patients were counselled by their physician to reduce sitting time [26]. The present study was the first to our knowledge to address RDs’ intention regarding their PA and SB counselling and therefore more research in this area is also needed.

More research is needed to further examine RDs’ attitude toward PA and SB counselling. While some participants had a positive attitude about the effectiveness of RD counselling on PA and SB (i.e., feeling that RD counselling in these areas is effective in increasing patients’ PA and decreasing their SB), their belief about effectiveness was contingent on a number of factors such as time frame for behaviour change. Consistent with the current findings, a systematic review found that primary care providers (including nurses, nurse practitioners, physicians, and physicians’ assistants) were unsure of the effectiveness of their PA counselling [51]. Focus group findings in the Spidel et al. [19] study indicated strong support from RDs for incorporating PA counselling into RDs’ daily practices, but RDs’ views on the effectiveness of their counselling were not discussed. To determine the effectiveness of PA and SB counselling, intervention research involving rigorous research designs are needed.

Results from the literature are mixed regarding injunctive normative beliefs among HCPs and HCPs in training. RDs in past studies agreed that PA counselling or promoting active living is part of their role as an RD [19, 20, 25], which is consistent with the current results. Participants in the current study felt that all HCPs, including RDs, should counsel patients on PA and SB. Similar to Spidel et al.’s [19] findings, participants in the present study felt that they should provide general counselling about PA only and that exercise prescription is outside their scope of practice. However, whereas many current participants felt that other HCPs expect them to counsel on PA, a study of primary care residents found that residents rated obesity, nutrition and PA counselling norms (including what was expected of them and the importance that their peers and profession placed on obesity, nutrition and PA counselling) as low [52]. Some current participants believed that other HCPs did not expect them to provide PA and SB counselling, which is similar to Spidel et al.’s [19] finding that RDs had concerns about how other HCPs (as well as the public) would perceive their counselling in this area.

Regarding descriptive normative beliefs, greater communication among HCPs is warranted. Although many participants had not asked other RDs about their practices, most believed that other RDs were counselling on PA, and many felt the same was true for SB. Past studies have not addressed communication among RDs regarding PA and SB counselling, though previous research suggested a need for increased communication and collaboration between RDs and PA specialists [19]. A study of the promotion of healthy eating among exercise specialists suggested that there was a need for increased collaboration between RDs and exercise specialists so that RDs could support exercise specialists in their role as healthy eating promoters as well [53].

Participants were divided in their opinion of what is expected of them by other HCPs. In order to improve support between HCPs, particularly in team-based settings, specific improvements in communication may be required. If RDs know what is expected of them pertaining to PA and SB counselling and also know what other RDs’ counselling practices are in these areas, they could feel more supported in their role regarding PA and SB counselling. This is important considering that previous research among primary HCPs revealed that subjective norm can have a meaningful influence on behavioural intention, and subsequently, behaviour [54]. Also, greater communication could elicit conversations regarding division of responsibility in terms of PA and SB counselling among RDs and other HCPs in FHTs.

Findings from the literature are mixed regarding HCPs’ perceived behavioural control. Perceived behavioural control is often considered comparable to self-efficacy [36]. Whereas almost all participants in the present study were confident in basic PA counselling only, a previous study found that self-efficacy related to PA counselling is relatively low among RDs [19] and a recent review found this to be true for other HCPs as well [54]. To our knowledge, there has been no previous research regarding HCPs’ self-efficacy related to SB counselling. The current research was the first of its kind to study RDs in FHTs and therefore participants identified facilitators that were likely unique to the FHT and articulated how the FHT setting (a collaborative team of HCPs with increased access to patients and patients’ health information) made it easier to provide PA counselling. Time was discussed as a facilitator to counselling in the present study but identified as a barrier in previous studies [19, 20]. Most participants in the present study stated that they had ample time with patients during appointments to cover PA counselling. The discrepancy in these findings could be attributed to the different practice settings for participants in the present study compared with past studies. In the studies that found time to be a barrier, RDs were working in a variety of practice settings [19] or in mostly clinical settings [20]. Perhaps RDs working in clinical settings have less time to counsel patients on PA, as it is likely that managing acute nutrition-related issues takes precedence.

Past studies addressing RDs’ PA counselling found that the main barriers to counselling included lack of protocol surrounding RDs’ PA counselling [20], time constraints [19, 20], concerns about public or other HCPs’ perceptions of RDs counselling on PA [19], and lack of knowledge or training in PA counselling [19, 25]. Also, to our knowledge, there are no previous studies addressing facilitators and barriers that influence HCPs’ SB counselling. Though participants in the present study did not mention the term “lack of protocol” when discussing barriers to PA counselling, RDs’ scope of practice was identified as a barrier. Some participants in the current study felt that PA assessment and exercise demonstration are outside their scope of practice. While RDs in a previous study reported PA monitoring to be outside their scope of practice or role [19], this was not mentioned by participants in the present study.

In particular, many current participants felt that exercise prescription is outside their scope of practice. This aligns with a recognised PA resource, specifically developed for RDs by both nutrition and exercise professionals. The resource suggests that RDs can provide general PA guidance (e.g., counsel about the PA recommendations; discuss goal setting related to PA), based on national PA guidelines, to patients but RDs require fitness certification to assess fitness and prescribe exercise [55]. In previous studies, it was found that out of the four domains of exercise prescription (frequency, intensity, duration, and type), RDs addressed frequency most often and intensity least often [20, 25]. Though the interview guide did not address these domains specifically, the current participants discussed that they were less comfortable counselling patients about higher intensity exercises, but they counselled patients using the PA guidelines, which includes intensity (i.e., 150 min of MVPA per week in at least 10-min bouts). George et al. [20] found that RDs felt that lack of protocol for PA counselling negatively affected their counselling. If RDs were to have a clear protocol to follow when counselling patients on PA, they may feel more confident that they are counselling patients within their scope of practice. Increased HCP self-efficacy has been found to have a positive influence on the level of PA counselling among RDs [54], and the same may be true for SB counselling. In terms of SB counselling and RDs’ scope of practice, though participants in the present study felt that most aspects of SB counselling fit within their scope of practice, there is no previous research in this area to compare these findings to.

The organisation of HCP roles in Canada influenced these RDs’ perceptions of PA and SB counselling. RDs in Canada are regulated by provincial legislation, while standards for education/training programs are national [56, 57]. Currently, the national competency standards do not include specific standards/competencies regarding PA and SB and counselling in these areas. Instead, they are quite general in terms of encouraging overall health, which would encompass basic PA counselling. In Ontario, the College of Dietitians of Ontario regulates practice through Controlled Acts and each health profession is under a different College [58]. Neither diet nor PA counselling fall under any of the Controlled Acts, so any HCP can counsel in these areas. If PA and SB interventions are considered to require specific skills, then further development of education and regulations within different health care systems to define scope of practice for different HCP groups will be needed.

Providing more education about PA and SB counselling to HCPs is warranted. Past studies consistently cited knowledge as a barrier to PA counselling among RDs [19, 25] and other HCPs [54]. In a study of PA counselling among RDs [20], knowledge was not explicitly stated as a barrier but nonetheless only a third of interviewed RDs received PA or PA counselling education in the last 5 years. Similarly, in the present study, participants discussed lack of PA and SB knowledge as an obstacle to counselling in these areas. Conversely, in a recent review, knowledge and training were facilitators to PA counselling among some HCPs [54]. Some participants in the current study intended to pursue further education in PA, SB, and counselling in these areas. There is some related past research regarding RDs’ perceived needs to facilitate further PA counselling. In a UK-based, survey study, McKenna et al. [25] found that almost all RDs wanted further education in PA and counselling in this area, preferably in the form of a day course. Similarly, in a focus-group-based study conducted in Alberta, Canada, Spidel et al. [19] found that RDs wanted more education about exercise physiology, PA assessment, and ways to monitor PA.

There are strengths and limitations of the present study. A qualitative approach was appropriately used to obtain a deeper understanding of the unexplored research topic regarding FHT RDs’ beliefs and behaviours related to counselling patients on PA and SB. There may have been selection bias in that RDs who are interested in PA and SB may have been more likely to volunteer to participate in the study. Also, the sample size was small (20 participants), though samples in qualitative research are often small. Nonetheless, theoretical saturation was reached during thematic analysis and some common themes were identified. Multiple authors identified themes from the audio-recorded and transcribed interviews to yield accurate results. However, the findings cannot be generalized to all RDs working in team-based primary care throughout Ontario. This exploratory study can provide direction for a subsequent qualitative study of FHT RDs sampled from across Ontario.

The issue of whether the research purpose was examined sufficiently by using TPB also warrants mention. TPB is a widely used theory that examines attitude, subjective norm, and perceived behavioral control, as key determinants of behavioural intention and ultimately behaviour. In addition to these three types of beliefs, other factors were examined in that subjective norm addressed the interpersonal component and perceived behavioural control addressed some environmental factors that made it easy or difficult to counsel patients on PA and SB. Also, utilizing theory when analyzing the qualitative data might initially appear as a limitation based on the premise that themes were not identified inductively. However, an inductive approach was actually used in that TPB informed which topics were addressed in the semi-structured interview guide (specifically, theoretical constructs that were of interest) and then, within that structure, themes for each TPB construct were identified inductively.

## Conclusions

RDs in FHT settings are uniquely positioned to counsel patients on PA and SB, as most of their counselling is focused on positive lifestyle change and similar techniques are used across different behaviour change interventions. In addition to this, RDs are currently core members of FHTs, in contrast to fitness experts. The results of this study indicated that participants provided general PA and SB counselling to patients, had both positive and negative feelings about it, expected HCPs to provide it, and identified factors that facilitate it (e.g., FHT dynamics) or impede it (e.g., only basic PA counselling is within their scope of practice; lack of knowledge about PA and SB). RDs and university students enrolled in a nutrition/dietetics program (i.e., dietitians in training) in Canada can independently use existing resources to increase their knowledge, enhance their positive attitude (e.g., by learning about the benefits of PA), and improve their counselling skills regarding PA. For example, the “Physical activity guidelines for Americans” is an evidence-based resource for health professionals to design and implement PA programs [7]. Also, “A physical activity toolkit for registered dietitians: Utilizing resources of exercise is medicine” is a resource for RDs to provide PA guidance to patients within their current scope of practice [55]. This toolkit discusses a decision analysis tool that is intended to assist RDs in determining which aspects of counselling on PA and SB fit within their current scope of practice. Additionally, to increase confidence and reduce possible risk to patients, RDs could consider encouraging patients to engage in light-intensity PA, considering that a systematic review found that light-intensity PA was associated with positive health outcomes among adults [59]. Specifically, light-intensity PA (measured by accelerometer) was inversely associated with all-cause mortality risk and favourably associated with cardiometabolic risk factors, after controlling for MVPA. RDs’ SB counselling could include strategies such as: interrupt prolonged sitting at work and home with standing or PA breaks; replace sitting time with light-intensity PA (e.g., walking slowly; light household chores) and MVPA; use a smartphone, watch, or electronic calendar to send reminders to stand up, stretch, or walk; stand or walk when talking on the phone; walk to co-workers’ offices to communicate in person instead of by phone or email; schedule walking meetings; and use sit-stand desks.

Also, RDs and university nutrition/dietetics students can explore the option of completing a fitness certification program to acquire knowledge and skills related to fitness assessment and exercise prescription, as continuing education. This would enable them to assist patients beyond providing general PA guidance when integrating PA and nutrition counselling in their practice. For example, Canadian Society for Exercise Physiology [60] has a certification program (which includes study and examination preparation resources) for persons with at least 2 years of university coursework in areas related to exercise science to become a certified personal trainer who can design and implement exercise programs for apparently healthy clients and clients with a stable health condition. Canadian Society for Exercise Physiology certified personal trainers have professional liability insurance to support their own scope of practice. This contrasts with the liability issue with RDs who are not certified fitness professionals assessing fitness and prescribing exercise, which was raised in the current study. As the role of RDs as PA counsellors continues to evolve, additional work by regulators and educators is likely needed to address how PA and SB services to patients should be organised to ensure maximal effectiveness and safety.

This study provides a strong foundation to develop a TPB-based, quantitative measure to assess behaviour, behavioural intention, attitude, subjective norm, and perceived behavioural control related to PA and SB counselling among a sample of RDs and other HCPs. Such a measure can then be used in a subsequent needs assessment to identify and track specific needs of RDs and other HCPs to successfully counsel patients on PA and SB.

## Supplementary Information

**Additional file 1.** Interview Guide.

## Data Availability

The dataset generated and/or analysed during the current study is not publicly available because the transcripts of the interviews could be traced back to the participants, personal confidentiality may be compromised, and there was no provision in the approved research ethics protocol to make the raw data publicly available.

## Abbreviations

FHT: Family health team
HCP: Health care professional
MVPA: Moderate-to-vigorous physical activity
PA: Physical activity
RD: Registered dietitian
SB: Sedentary behaviour
TPB: Theory of planned behaviour
