# Supplementary material for: Registered dietitians’ beliefs and behaviours related to counselling patients on physical activity and sedentary behaviour from a theory of planned behaviour perspective
Source: BMC Nutr. 2020 Nov 30;6:66. doi: 10.1186/s40795-020-00392-1 (PMC7702673; doi:10.1186/s40795-020-00392-1)
Supplement: Supplementary file 1 — Additional file 1. Interview Guide. [file 40795_2020_392_MOESM1_ESM.docx]

| Supplementary file: Interview Guide | |
| --- | --- |
| Question | TPB construct |
| Can you share with me your experiences, if any, on counselling patients on PA in the FHT setting?  What are your experiences, if any, on counselling patients on SB in the FHT setting? | Behaviour |
| Can you share with me your intention, if any, to counsel patients on PA in the future?  Can you share with me your intention, if any, to counsel patients on SB in the future? | Behavioural intention |
| Overall, do you feel that RDs counselling patients on PA is an effective or ineffective way to improve this behaviour? Why?  Overall, do you feel that RDs counselling patients on SB is an effective or ineffective way to improve this behaviour? Why? | Attitude |
| As an RD, what do you feel other HCPs expect of you in terms of PA and SB counselling?  Do you think that other RDs in FHTs counsel patients regularly on PA and SB, and why or why not? | Subjective norm |
| What are the circumstances or things in the FHT setting that make it easy or difficult for you to counsel patients on PA?  What are the circumstances or things in the FHT setting that make it easy or difficult for you to counsel patients on SB? | Perceived behavioural control |
